# Outcomes of drain versus no drain in total knee arthroplasty: a retrospective cohort study

**DOI:** 10.1007/s00264-023-05946-z

**Published:** 2023-08-23

**Authors:** Anas Albasha, Loay A. Salman, Ahmed Elramadi, Abedallah Abudalou, Ahmed Mustafa, Hasan Azzam Abu Hejleh, Ghalib Ahmed

**Affiliations:** 1 Orthopedics Department, Hamad General Hospital, Hamad Medical Corporation, PO Box 3050, Doha, Qatar; 2 Department of Orthopaedic Surgery, Surgical Specialty Center, Hamad Medical Corporation, Doha, Qatar; 3 Medical Intern, Hamad General Hospital, Hamad Medical Corporation, PO Box 3050, Doha, Qatar

**Keywords:** Total knee arthroplasty, Suction drain, Infection rate, Blood loss, Blood transfusion, Length of hospital stay

## Abstract

**Purpose:**

The use of suction drains in total knee arthroplasty (TKA) remains controversial. The aim of this study is to compare the outcomes of patients who received suction drains versus those who did not, focusing on blood loss, blood transfusion need, and length of hospital stay.

**Methods:**

A retrospective observational cohort study was conducted at a tertiary hospital between January 1, 2015, and December 30, 2019, and included 262 patients who underwent unilateral non-traumatic primary TKA and were over 18 years old. The Institutional Review Board (IRB) approved the study (MRC-02–20-278).

**Results:**

A total of 262 patients were included, with an age range of 47 to 91 years. Most of the included patients were females, 74.4% (195). Hypertension was the most frequent risk factor, 67.6%, followed by diabetes. Of 262 patients, 156 (59.5%) received a drain. The drain group had significantly longer hospital stay, 30% longer tourniquet time, greater haemoglobin and haematocrit drops, higher count of transfused packed RBC units, and lower use of anticoagulants. Moreover, tranexamic acid (TXA) use (*n* = 106) in surgery reduced hospital stays, tourniquet time, drain output, and increased pre- and postoperative haemoglobin and hematocrit levels compared to no TXA group (*n* = 156) (*p* < 0.05, z-score reported).

**Conclusions:**

This study found that patients who received a drain had longer hospital stays and greater blood loss and transfusion rates compared to those who did not. The use of TXA in surgery was associated with improved outcomes and reduced overall complications.

## Introduction

The use of suction drains following total knee arthroplasty (TKA) remains debatable, with considerable variation in its use across different regions and hospitals. The frequency of total knee arthroplasty (TKA) procedures has been rising steadily in recent years. In the United States, for instance, the number of TKA procedures performed annually more than doubled between 2000 and 2010, with over 700,000 procedures conducted each year [1]. The total cost of managing complications after TKA has been estimated to range from $3,600 to $7,000 per patient [2].

Several studies advocated for the use of drain following TKA due to claims of reduced haematoma collection and associated wound complications, serous leakage at the wound, and translation to decreased postoperative limb swelling, reduced nidus for infection, as well as improved range of motion [3–5]. However, an increase in postoperative infection, blood loss, the need for blood transfusion, and length of stay have been associated with drain use, on the other hand [6–9].

Several studies have compared the use of drains versus no drains after TKA. One randomized controlled trial found that using a drain after TKA was associated with reduced postoperative blood loss and improved range of motion [10]. However, another randomized controlled trial found no difference in clinical outcomes between patients who received a drain and those who did not [11].

Therefore, the purpose of this study was to to evaluate the effectiveness and safety of using suction drains in TKA by comparing outcomes in patients who received drains versus those who did not. We hypothesized that there is no significant difference exists between both groups in terms of clinical outcomes and complication rate.

## Methods

This retrospective cohort study was approved by the Institutional Medical Research Centre (MRC-02-20-278) and was conducted at a single tertiary care center that holds accreditations from the Joint Commission International (JCI) and Accreditation Council of Graduate Medical Education-International (ACGME-I). The study adhered to the STROCSS guidelines [12] for reporting observational cohort studies. A retrospective review of electronic medical records of all adult patients who underwent primary TKA due to osteoarthritis at Hamad General Hospital between January 2015 and December 2019 was conducted. Power analysis was performed and a target sample size of 260 was set for this study.

The inclusion criteria for this study were patients aged 18 years and above who underwent primary TKA due to osteoarthritis. Exclusion criteria entailed patients with coagulopathy, traumatic total knee replacements, inflammatory arthritis, simultaneous bilateral total knee arthroplasty, malignancy, and revision TKA. Data collected included age, sex, surgery date, use of TXA, blood transfusion status (intra or post-operative), post-operative haemoglobin level, number of dressings done until the last follow-up, range of motion, and the rate of complications such as deep vein thrombosis and pulmonary embolism.

Total knee replacement surgeries were performed by multiple knee surgeons. The utilization of Tranexamic Acid (TXA) was found to be associated with the individual preferences of the operating surgeons. Certain surgeons adhered to a specific protocol involving the administration of 10-15 mg/kg TXA during the surgical induction and an additional 1 mg upon the deflation of the tourniquet. Conversely, other surgeons routinely chose not to employ TXA during the surgical procedures.

## Statistical analysis

Data was collected using a Microsoft excel online spreadsheet that was used by two authors independently. STATA version 17 software was used for data analysis. The normality of the distribution of the following continuous variables (Age, duration for drain removal, and drain output) was tested using Shapiro –Wilk W test (4 ≤ n ≤ 2000 observations) and Q–Q plot; proved not to have normal distribution; thus Mann–Whitney U-test was used to test the significance of difference for these continuous variables, and the chi-square tests for categorical variables.

## Results

### Patient’s demographics

In the study period from June 2016 to September 2020, a total of 262 cases underwent Total Knee arthroplasty. The majority were females, accounting for 74.4%, with ages ranging from 47 to 91 years old. Among the operated cases, 199 (76%) had at least two comorbidities, with hypertension being the most frequent, accounting for 67.6% of cases, followed by type 2 diabetes mellitus. Further details regarding the sample population are presented in Table 1. Regarding gender variations, both genders had similar baseline characteristics, as shown in Table 1. However, males tended to have lower comorbidity counts and lower BMI values. Nonetheless, 95% of the study population were overweight with a BMI over 25 kg/m2.

**Table 1:** Baseline characteristics of the study population

Parameter	Males	Females	*P*-value
Count	67 (25.6%)	195 (74.4%)	
Age in “years” (std. dev)	67.2 (9.3)	65.5 (7.6)	0.24
BMI	31.9 (5.1)	36 (6.1)	0.000*
Count of comorbidities	2.1 (1.3)	2.6 (1.2)	0.001*
#1. HTN	45 (67.2%)	132 (67.7%)	
#2. DM	33 (49.3%)	97 (49.7%)	
Hospital Stay (Days)	8.9 (5.6)	8.3 (5.2)	
Received TXA	29 (43.3%)	77 (39.5%)	0.67
Anticoagulation count	1.13 (0.42)	1.04(0.42)	0.10
Blood transfusion	7 (10.5%)	13 (6.7%)	0.30

### Drain versus No-drain

A surgical drain was applied in 156 patients (59.5%). The baseline characteristics of the patients, such as age, BMI, and comorbidities, were matched between the two groups, and no significant differences were found (P-value > 0.05). However, the group that received a drain had a significantly longer hospital stay (10.7 + -4.7 vs 5.4 + -4.6 days, P = 0.000), a 30% longer tourniquet time (111 vs 80 min, P = 0.000), a greater drop in haemoglobin (1.8 + -1.0 vs 1.2 + -0.7, g/dl P = 0.000) and haematocrit (5.4 + -3.0 vs 3.5 + -2.3,% P = 0.000), a higher average count of transfused packed RBC units (0.18 + -0.6 vs 0.04 + -0.2, units received P = 0.017), where each unit is of 250–300 ml per bag, and lower count of anticoagulants dose used (1.01 + -0.36 vs 1.13 + -0.50, units P = 0.025); i.e. daltaparin units. A detailed summary of the comparison between the drain and no-drain groups is presented in Table 2.

**Table 2:** Comparison of outcome variables, in terms of drain use. Hb: Hemoglobin. Hct: Hematocrit. SD: Standard deviation

Parameter	Drain used (*n* = 156)	No drain (*n* = 106)	*P*-value
*Independent*			
Age in years (SD)	66.6 (8.1)	65.0 (8.0)	0.14
BMI	34.7 (6.3)	35.6 (5.8)	0.22
Count of comorbidities	2.4 (1.3)	2.6 (1.2)	0.19
*Dependent*			
Hospital Stay (Days)	10.7 (4.7)	5.4 (4.6)	0.000*
Tourniquet time (mins)	111.8 (19.6)	80.8 (28.3)	0.000*
Hb (pre- & post-op) g/dl	1.8 (1.0)	1.2 (0.7)	0.000*
Hct (pre- & post-op) %	5.4 (3.0)	3.5 (2.3)	0.000*
Transfused packed RBC units (L)	0.18 (0.6)	0.04 (0.2)	0.017*
Count of Anticoagulation meds (dalteparin IU)	1.01 (0.36)	1.13 (0.50)	0.025*

^*^Statistically significant *P*-value

### Tranexamic acid (TXA) use

A sub-analysis based on tranexamic acid (TXA) use was conducted (Table 3). A total of 106 patients (40.6%) received TXA intraoperatively, based on surgeon’s preference and bleeding anticipation, and independent of drain use. Baseline characteristics such as age, BMI, and count of comorbidities were similar between the two groups (P-value > 0.05).

**Table 3:** Comparison of outcome variables, in terms of receiving the TXA treatment

Parameter	Received TXA (*n* = 106)	No TXA (*n* = 156)	P-value
*Independent*			
Age in years	64.96 (7.3)	66.60 (8.5)	0.14
BMI	35.1 (6.2)	34.99 (6.1)	0.63
Count of comorbidities	2.6 (1.3)	2.4 (1.2)	0.08
*Dependent*			
Hospital Stay (Days)	6.3 (4.8)	10.1 (5.1)	0.000* z = 6.6
Tourniquet used	90 (84.9%)	154 (98.7%)	
Tourniquet time (mins)	88.2 (30.4)	106 (24.2)	0.000* z = 4.6
Drain output (mls)	82.1 (167.9)	381 (263.7)	0.000* z = 9.3
Blood loss quantified	*n* = *55*	*n* = *141*	
Hb (pre- & post-op) g/dl	1.4 (0.6)	1.6 (0.98)	0.03* z = 2.2
Hct (pre- & post-op) %	4.95 (3.1)	4.06(2.1)	0.016* z = 2.4
Transfused packed RBC units (L)	0.08 (0.37)	0.15 (0.53)	0.32
Count of Anticoagulation meds (dalteparin IU)	1.21 (0.49)	0.96 (0.34)	0.000* z = -4.66
Post-op complications	0.019(0.14)	0.013 (0.11)	0.696

^*^Z score is reported only for those with significant *P*-value

*Hb* Hemoglobin, *Hct* Hematocrit, *SD* Standard deviation

In the TXA group (*n* = 106), there was a significant reduction in hospital stays (6.3 ± 4.8 days vs 10.1 ± 5.1 days, p = 0.000, z = 6.6), shorter tourniquet time (88.2 ± 30.4 min vs 106 ± 24.2 min, p = 0.000, z = 4.6), less drain output (82.1 ± 167.9 mls vs 381 ± 263.7 mls, p = 0.000, z = 9.3), and higher levels of pre- and postoperative haemoglobin (1.4 ± 0.6 g/dl vs 1.6 ± 0.98 g/dl, p = 0.03, z = 2.2) and haematocrit (4.95 ± 3.1% vs 4.06 ± 2.1%, p = 0.016, z = 2.4) compared to the no TXA group (*n* = 156). Additionally, TXA use was associated with a higher absolute count of anticoagulation doses (1.21 ± 0.49 vs 0.96 ± 0.34, p = 0.000, z = -4.66). However, there was no statistically significant difference in the occurrence of postoperative complications between the two groups (P-value = 0.696).

### Blood transfusion rate

20 cases had received blood transfusion (7.6%); with males 1.5 times more than females, as demonstrated in Table 2. Binary logistic regression demonstrated a statistically significant association between the following variables and the rate of blood transfusion: the BMI (Odds Ratio: 0.89, p-value = 0.015), the Pre-operative haemoglobin readings (OR: 1,p-value = 0.021), and the drain output (OR: 5.59, p-value = 0.03). This association is then modified when factoring for the use of tranexamic acid; among those with “no tranexamic acid”; the association of the BMI (OR: 0.90, p-value = 0.05), the pre-operative haemoglobin readings (OR: 0.61, p-value = 0.044) has persisted, while became insignificant for the Drain output (OR: 1, p-value = 0.104), however, in the group where “tranexamic acid” was used; the significance of all of them disappeared.

## Discussion

Total knee replacement (TKR) is a common surgical treatment for patients with end-stage knee osteoarthritis, as it has been found to significantly reduce pain, improve patient quality of life, and function [13–15]. However, efforts have been made to reduce post-operative blood loss, which can reach up to 1000–1500 ml, including hidden blood loss and intraoperative bleeding, as well as the need for blood transfusion and to prevent surgical and deep wound infections post-TKR [16–18].

A previous nationwide study reported that TKR is one of the top 10 most frequent procedures requiring transfusion in the USA, and blood transfusion is associated with serious complications, such as wound infection, an increase in hospital stay, in-hospital mortality [19–21], allogeneic blood reaction, and deep venous thromboembolism. Therefore, reducing the need for blood transfusion is essential to prevent such complications.

Several previous studies [17, 22, 23] have reported that closed suction drainage (CSD) was associated with increased total blood loss in TKR. A Level I-2 study [17] found that closed suction drainage increased the transfusion requirements after elective knee arthroplasty and had no major benefits, and Al-Zahid et al. [22] did not support the use of closed suction drains in primary elective TKR. In addition, Bjerke-Kroll et al. [24] reported that CSD increased the number of allogeneic blood transfusions in unilateral primary TKR, possibly due to blood loss in the drain group. The apparent preventive effect for the BMI and “pre-operative Haemoglobin” on the rate of post-operative blood transfusion might be explained by the fact that both variables are identified “pre-operatively” and accounted for in prospectively manner; while the drain output only identified in retrospective and post-operatively.

Similar to our findings, D Yang et al. [25] conducted a retrospective study on 200 patients who received TKR and found that the use of a drain after primary TKR had no clinical benefit, even after tranexamic acid had already been administered during the treatment. H Xu et al. [23] also found, in a retrospective cohort study, that the use of a drain was associated with a higher transfusion rate and a longer length of hospital stay (LOS) in patients undergoing routine primary TKR, and he did not recommend the use of a drain post-TKR. Sharma et al. [26] and Bjerke-Kroll et al. [24] reported that the LOS was longer in the drain groups compared to the no-drain groups after TKR.

One of the catastrophic and devastating complications post-TKR is wound complications, especially wound infections, as superficial wound infection can develop into deep wound and joint infection if not treated properly [20]. Willemen et al. [27] conducted a prospective randomized controlled trial that enrolled 41 patients who underwent TKR and showed that bacteria cultures of all drain tips cut off at postoperative 24 h were negative, while five of 21 drain tips indwelling for 48 h after operation developed positive culture results (all bacteria were Staphylococci), indicating that closed suction drain could be the source of retrograde infection and the source of devastating complications in TKR.

## Conclusion

This study demonstrated that the use of suction drains in primary TKA was associated with longer hospital stays, increased blood loss, and higher transfusion requirements. Moreover, the use of TXA during surgery was found to be associated with better outcomes, including shorter hospital stays, decreased blood loss, improved haemoglobin and haematocrit levels, and less overall postoperative complications. However, the routine use of TXA in knee arthroplasty remains controversial and this finding should be applied in context due to potential confounders, including various patient’s and surgical factors. Further research may be needed to determine the optimal approach to managing blood loss in TKA.

## Data Availability

Happy to provide access to data (coding) upon request.
